# Gazing Strategies among Sentinels of a Cooperative Breeder Are Repeatable but Unrelated to Survival

**DOI:** 10.3390/biology13060458

**Published:** 2024-06-20

**Authors:** Guy Beauchamp, Sahas Barve

**Affiliations:** 1 Independent Researcher, Montréal, QC, Canada; 2 Archbold Research Station, 123 Main Dr., Venus, FL 33960, USA; sbarve@archbold-station.org

**Keywords:** birds, group size, personality, predation risk, vigilance

## Abstract

**Simple Summary:**

Animals can detect threats through their vigilance. Many studies indicate that traits such as vigilance are repeatable among individuals over time suggesting differences in the ways individuals respond to risk. Little is known about individual consistency in the ways vigilance is achieved from one moment to another and whether among-individual differences in vigilance are related to survival. Using sentinels of a cooperative breeder, the Florida scrub-jay (*Aphelocoma coerulescens*), we examined the occurrence of stable individual patterns of vigilance during sentinel bouts and their association with survival. During sentinel bouts from vantage points, Florida scrub-jays turn their heads from side to side to monitor their surroundings for threats such as intruding neighbours or predators. Using data from three field seasons, we found that the head-turning frequency was repeatable in breeders but not in younger birds and was not clearly associated with survival. Younger birds typically have less experience with threats, which might mitigate against the occurrence of consistent individual differences at that age. The lack of association between the head-turning frequency and survival was not expected. Future studies are needed to validate this crucial assumption of vigilance in animals.

**Abstract:**

Vigilance is a common behavioural adaptation to increase the chances of detecting predators before it is too late to escape. Behavioural traits are often repeatable among individuals over the long term, suggesting differences in personality. Earlier studies have documented individual consistency in the time allocated to vigilance. However, little is known about individual consistency in the ways vigilance is achieved from one moment to another and whether different patterns of vigilance among individuals are associated with survival. We aimed to determine whether sentinels of a cooperative breeder showed individual consistency in their vigilance and if individual variation was related to annual survival. During sentinel bouts from vantage points, Florida scrub-jays (*Aphelocoma coerulescens*) turn their heads from side to side to monitor their surroundings. Over three field seasons, we found that the head-turning frequency was repeatable in breeders but not in juveniles or non-breeding helpers. The moderate repeatability in breeders was not related to survival. Our results suggest that the head-turning frequency in sentinels of the Florida scrub-jay is repeatable in breeders but not in less experienced juveniles or helpers and, therefore, likely becomes more repeatable as individuals age. The assumption that individual variation in vigilance is related to survival was unsupported in our study and requires further study.

## 1. Introduction

Predation is a major source of mortality in vertebrates [1]. These animals have evolved various strategies to reduce predation threats. Some strategies involve morphological adaptations like camouflage to reduce encounter rates with predators [2]. Other strategies rely on changes in behaviour such as living in groups and vigilance to reduce predation risk [3,4]. Vigilance, in particular, represents an investment in time to increase the chances of detecting predators before it is too late to escape [5,6]. Investment in vigilance often comes at a cost as it reduces the time available to perform other fitness-enhancing activities such as resting and feeding.

In group-living species, more eyes and ears are available to detect predators, and individuals can reduce their own investment in vigilance at no increased risk to themselves [7]. To obtain the full benefits from collective vigilance, group members are expected to initiate their vigilance bouts independently from one another [8]. However, in some cases, individuals are more likely to become vigilant when their neighbours are more vigilant themselves, leading to the synchronization of vigilance at the group level [9,10,11,12]. In other cases, individuals are more likely to become vigilant when others are less vigilant, leading to the coordination of vigilance [13,14,15]. The level of surveillance achieved in a group with coordinated vigilance is more constant than when vigilance is synchronized or independent, providing a clear survival advantage [16,17,18]. The coordination of vigilance has been documented in groups with sentinels [19,20,21]. In such species, one or more sentinels monitor their surroundings from vantage points and individuals in the group take turns as sentinels during foraging [22].

Can a trait like anti-predator vigilance be individually consistent? For many traits, behaviour is repeatable over time at the individual level, suggesting that variation among individuals at any given time is not simply the result of individual variation in state variables that fluctuate more or less randomly from time to time such as hunger [23]. Consistent traits over time can be considered personality attributes and may even be correlated with other traits to form behavioural syndromes [24]. Consistent variation among individuals in vigilance was not anticipated in earlier models of vigilance. For instance, in group-living species, where vigilance is considered the outcome of an evolutionary game between group members, individuals that stray from the optimal level of vigilance predicted for the group were expected to pay a survival cost [25]. However, research on personality has emphasized the occurrence of consistent differences in risk taking among individuals with some individuals more tolerant of risks than others [26,27,28]. As vigilance represents a form of risk management, it might be expected that some individuals are consistently more vigilant than others.

The empirical evidence thus far supports the existence of consistent differences in vigilance. Individual differences have been documented in mammals [29,30,31,32,33,34,35,36,37] and in birds [26,38,39,40]. In addition, individuals can also vary consistently in the way vigilance is adjusted to gradients in predation risk [26,29,41] although there are exceptions [31,34,40]. Despite the mounting evidence, it is still unclear whether consistency in vigilance is common in animals, how such consistency develops with age, and how it is maintained in the population.

Documenting consistency in vigilance is challenging as it requires long-term studies with repeated observations on the same subjects to exclude the possibility that vigilance is consistent simply because the environment that shapes vigilance does not change quickly [42]. In addition, as vigilance is expected to vary with a host of ecological factors, such as group size, dominance status within the group, hunger, and spatial position, it is important to control for such variables when investigating consistency in vigilance. For instance, vigilance often tends to be higher at the periphery of groups [43,44]. If individuals vary consistently in the spatial position they occupy in groups, perhaps in relation to dominance status within the group, then vigilance will also vary consistently among individuals if spatial position is ignored. Similarly, hungrier individuals are expected to be less vigilant than others [25], and if hunger levels are consistent among individuals, this might lead to consistency in vigilance unrelated to personality.

Most of the studies on vigilance consistency have focused on the allocation of time to vigilance. How vigilance is achieved from one moment to another is also just as important to predator detection as time investment and might also be a target to examine consistency in vigilance. Because animal eyes typically have a limited field of vision, animals need strategies to monitor their surroundings in order to detect predators efficiently [45]. For instance, animals can turn their heads in different directions to monitor different parts of their surroundings [46,47,48]. Animals can turn their heads at different rates to monitor different areas more frequently or more thoroughly depending on the perceived predation risk. It is not clear whether the gazing strategies that emerge from head turning are consistent among individuals. In the only study we could find, the head-turning frequency was not consistent in laboratory birds [49].

Few studies have related consistent variation in behaviour among individuals to survival using appropriate statistical methods [50]. As far as we are aware, only one study examined the relationship between variation in vigilance among individuals and survival and found no significant association [51]. If the main purpose of vigilance is to evade predation, variation among individuals in vigilance should be related to survival. One possibility is that the various means to achieve vigilance are alternatives with similar survival values. Another possibility is that different gazing strategies are not equally successful in terms of survival against predators, but fitness deficits are compensated in other contexts such as reproduction [24].

**Figure 1:**
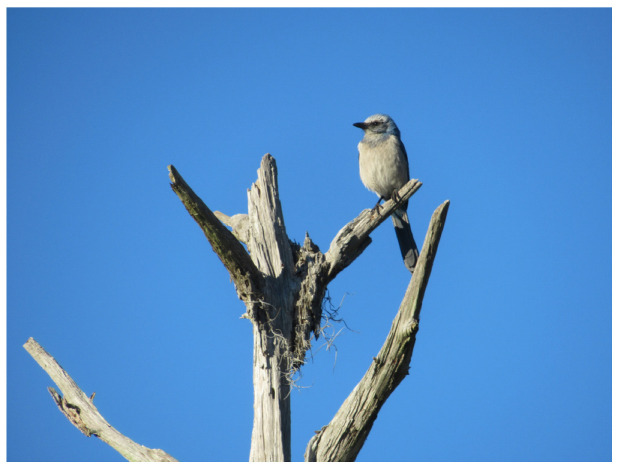
Florida scrub-jay sentinels monitor their surroundings from vantage points above the cluttered foraging habitat (picture by G.B.).

In this study, we examined consistency in the gazing strategies of sentinels in a cooperative breeder, the Florida scrub-jay (*Aphelocoma coerulescens*). The Florida scrub-jay is a cooperative breeder living in all-purpose territories year-round [52]. Offspring can remain in the territory of their parents for several years and assist the family in parental care, territory defence, and predator detection as non-breeding helpers. Breeders are extremely sedentary, which makes it easy to determine the fate of these individuals. Florida scrub-jays forage for invertebrates and acorns on or near the ground among dense vegetation. Sentinels monitor their surroundings from vantage points above the cluttered foraging habitat [19,53] and alert family members foraging nearby when they detect intruders from other territories or predators like hawks (Figure 1).

Sentinels move their heads from side to side to monitor different parts of their surroundings, and the frequency of head turning has been related to the perceived predation risk. When facing a higher risk of predation, shorter looks in many directions might allow individuals to monitor a greater area more quickly, minimizing the chances of failing to detect an approaching threat from any direction. Indeed, sentinels turned their heads more frequently in smaller, more vulnerable groups [54]. Sentinels with less experience with predation threats, such as juveniles or helpers, also turned their heads more frequently [54]. The head-turning frequency also decreased when multiple sentinels were present at the same time, which is compatible with risk dilution [55]. The height at which sentinels monitor their surroundings might also affect head turning as higher sentinels can probably detect threats farther away and could afford to spend more time looking in any one direction [56]. The head-turning frequency in sentinels might thus be a key factor in the effectiveness of vigilance.

The duration of sentinel bouts may be related to hunger levels [57]. However, gazing strategies during a sentinel bout are not expected to vary with hunger as head turning is not related to food acquisition. Sentinels are not competing with other group members for resources, so their vigilance can be fully dedicated to threats outside rather than within the group. Without interference from state variables such as hunger or dominance, it might be easier to document consistency in vigilance. We sought to determine whether head turning is individually consistent in sentinels of the Florida scrub-jay. Consistency, if present, should be more likely in individuals with more experience with predation threats like breeders, which are less likely to alter their vigilance from one moment to another due to imagined threats [58]. We also sought to determine whether consistent head turning, if present, is associated with survival.

## 2. Materials and Methods

This study was carried out at Archbold Biological Station located in South Central Florida, USA (27.1° N, 81.2° W). The vegetation at the station is characterized by small scrub oaks (*Quercus* spp.) with scattered pine trees. Nearly all Florida scrub-jays in the research tract at the station are banded, and their sex, age, and social status are known. The population is monitored monthly to determine the fate of marked birds. We carried out observations during the winter months from 2022 to 2024, typically from 7 to 11 a.m. when sentinel behaviour was most frequent.

One of us walked along the many trails of the Station to locate foraging groups with sentinels. Sentinel behaviour was recorded with a video camera typically at close range (from 5 to 15 m) as the birds were accustomed to the presence of observers. Focal observations were scheduled to last 5 min unless the sentinel departed. At the beginning of an observation, the number of Florida scrub-jays present in the vicinity, including the sentinels, was recorded to obtain the group size. Unique combinations of coloured bands on each bird allowed us to identify the focal subjects. Sentinel perch height was evaluated visually in increments of about 1 m. We avoided observations during encounters between neighbouring groups as sentinel behaviour was often disrupted. Over the three field seasons, we obtained 253 videos from 109 unique breeders and 104 videos from 66 unique juveniles or helpers.

Playing each video at low speed, we extracted the number of detectable head movements and divided the total number of movements by focal observation duration to obtain the head-turning frequency. We established survival using two methods. First, we set day 1 for the survival period at the mid-March census of 2022 or 2023. We then determined whether each focal bird survived the next twelve months. As the last census for this study occurred in mid-March 2024, it was not possible to examine survival for the 2024 cohort with this method. Using data from monthly censuses, we also counted the number of months from the first observation of a focal bird until death. In all cases, we determined that a bird was dead if absent from two censuses in a row. Focal birds still alive at the mid-March census of 2024 were considered censored.

To determine the repeatability of head turning in sentinels, we used the intra-class correlation coefficient (ICC) obtained from a linear mixed model with sentinel ID as a random effect and group size, sex, age, presence of other sentinels, and perch height as the fixed effects. We carried out analyses separately for breeders and non-breeders. For the non-breeders, we pooled juveniles and helpers because repeated measurements were not as frequent for this class of individuals. ICC and the percentage of variation explained by the fixed effects were obtained using the *rptR* package (v. 0.9.22) in R [59].

For the correlation between head turning and survival, we used two approaches. We restricted these analyses to breeders as the fate of helpers was more difficult to determine due to possible emigration outside the research tract. For the first approach, we used a bi-variate model, the suggested approach to establish correlation between traits in personality research [42]. The bi-variate model has two components: the head-turning frequency and annual survival. The bi-variate approach jointly analyzes each dependent variable as a function of a set of independent variables, which makes it possible to obtain the correlated residual variances of the two traits not explained by the independent variables. The bi-variate approach produces the correlation between the two traits based on among-individual variation to determine whether individuals with overall higher frequencies of head turning are more or less likely to survive the year. The survival component was based on a mixed logistic regression with Bernoulli errors, including sentinel ID as a random effect and sex, age, and group size as fixed effects. Age was considered here as annual survival declines for older individuals in this species [60]. The dependent variable was annual survival (yes or no) each year. Only the first two years of this study could be included for the bi-variate survival analysis as mentioned earlier. For the head-turning frequency component, we used a linear mixed model with Gaussian errors, including sentinel ID as a random effect and group size, sex, age, presence of other sentinels, and perch height as fixed effects. The bi-variate model was fitted in a Bayesian framework in R with the *brms* package (v. 2.21.0) [61]. Briefly, we used two chains running for 10,000 iterations with a burn-in of 5000 iterations. The Gelman-Rubin statistic was used to assess convergence and mixing of the chains with potential scale reduction factors all less than 1.01.

The second approach took advantage of the information about monthly survival. We used Cox’s survival analysis with time to death in months as the dependent variable considering subjects still alive at the end of the last census censored. With this approach, it was possible to use data from all three field seasons. We used BLUP estimates from the repeatability analysis described earlier to distinguish between sentinels with head-turning frequencies above the median and below the median. Survival was compared between the two classes of sentinels. The survival analysis was run using the *coxme* package (v. 2.2-20) in R [62]. In all models described above, quantitative independent variables were scaled before analysis.

## 3. Results

Group size ranged from 2 to 9 with a median of 4. Sentinels monitored their surroundings at heights ranging from 0.1 m to 25 m with a median of 4 m. Focal observation duration lasted a median of 98 s.

Repeatability was moderate for breeders (R = 0.244, 95% credible interval (CI): 0.083, 0.393), and the independent variables explained 16.8% of the variation in the head-turning frequency. Repeatability was slight for juveniles and helpers (R = 0.095, 95% CI: 0, 0.415), and the independent variables explained 31.4% of the variation in the head-turning frequency.

**Figure 2:**
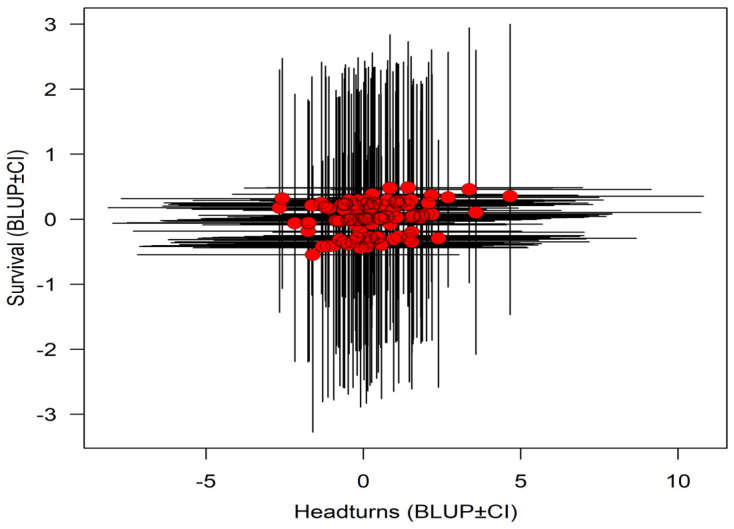
Bi-variate association in breeders between annual survival and head-turning frequency in sentinels of the Florida scrub-jay. Mean estimates are shown with red dots and 95% credible intervals are shown with black lines.

The bi-variate model revealed no clear among-individual correlations between annual survival and the head-turning frequency for breeders (r = 0.07, 95% CI: −0.92, 0.95, Table 1, Figure 2). None of the independent variables was clearly associated with the variation in annual survival (Table 1). The head-turning frequency decreased in larger groups and when sentinels were perched at greater heights. In addition, the head-turning frequency decreased when other sentinels were present (Table 1). The head-turning frequency in breeders was related neither to sex nor age (Table 1).

**Figure 3:**
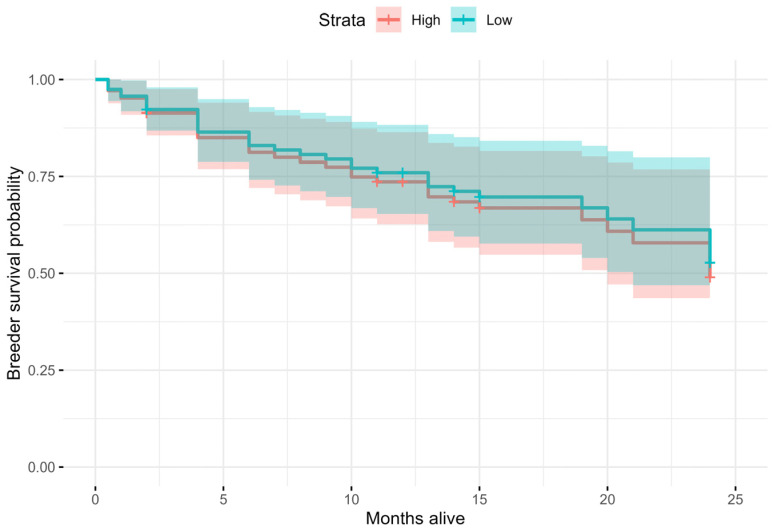
Cox survival analysis of the association between breeder survival in months and head-turning frequency in sentinels of the Florida scrub-jay. The plot shows the predicted survival proportion (95% confidence intervals) for sentinels with head-turning frequencies above (High) or below (Low) the median.

For the Cox survival analysis restricted to breeders, we recorded 34 mortalities over the two years. Using BLUPs from the repeatability analysis, the hazard of dying decreased by a factor of 0.90 (95% confidence interval: 0.46, 1.76) when the head-turning frequency for a sentinel was below rather than above the median, a non-significant effect (*p* = 0.7; Figure 3).

## 4. Discussion

Head-turning frequency in sentinels of the Florida scrub-jay varied as a function of factors like group size and perch height. These results fit with the idea that the head-turning frequency is associated with perceived predation risk [49,54,55]. The head-turning frequency also varied systematically among breeders but not among the younger juveniles and helpers. The moderate repeatability that we documented in breeders was not related to annual survival or to the number of months before death.

The individual consistency in the head-turning frequency in breeders supports findings from other studies showing that measurements of vigilance can vary systematically among individuals through time [26,29,34,35,39,40]. Our results indicate that individual consistency can be found not only in the overall allocation of time to vigilance but also in the way vigilance is achieved from one moment to another. Our estimate of repeatability for vigilance, a value of 0.244, is lower than what is typically reported for behavioural traits (around 0.4, [63]). Repeatability estimates obviously can vary depending on the length of studies and whether they take place in the laboratory or in the field. Individual consistency in sentinels was, at best, moderate despite the fact that we controlled for many important variables known to influence vigilance, such as group size, and that we could exclude the influence of state variables, such as hunger and dominance. Unexplained variation among individuals in the frequency of head turning is probably related to factors that act on a small time scale and are not consistent through time. Potential factors include, for instance, the recent history of encounters with predators [64] or rapid changes in weather variables such as light levels or wind that affect the ability to detect predators [65]. Despite significant repeatability, sentinel vigilance appears to be a largely labile trait in breeders of the Florida scrub-jay.

The lack of experience with predation threats might explain why repeatability was weaker in younger birds. In many species, young individuals need time to respond to and produce alarm calls appropriately [66]. Young birds are probably more influenced by factors that fluctuate more or less randomly on a small time scale. These factors are not necessarily related to predation risk but are perceived as such by inexperienced individuals. Therefore, their evaluation of predation risk is more likely to fluctuate from moment to moment, thus masking any individual consistency in vigilance. In a study looking at flight initiation distance, an anti-predator trait, repeatability was also lower in juveniles [67]. Our results suggest that vigilance, if measured in younger individuals, would be poorly correlated with their vigilance as adults, as has been found in other studies [68]. It is not clear when repeatability becomes established in sentinels of the Florida scrub-jay. More data on vigilance in helpers, the transitional stage between juveniles and breeders, is necessary to address this issue. In general, the development of sentinel behaviour is poorly known [69].

We did not find an association between the head-turning frequency and annual survival or survival from month to month in sentinels of the Florida scrub-jay. Very little work has examined the association between vigilance behaviour in general and survival using among-individual variation [51]. Other studies have found that lower vigilance is associated with lower survival [70,71,72] but did not use among-individual variation in survival or vigilance to establish the correlation, which can lead to spurious associations [73]. Other anti-predator traits like flight initiation distance are also typically moderately repeatable [74] and have been linked to survival in some cases [75,76] but not in others [77]. Low repeatability, as we found here for the head-turning frequency, makes it more challenging to document an association between behaviour and survival. Beyond methodological issues, it is perhaps the case that different frequencies of head turning are equivalent in terms of predator detection and thus have similar survival values. This idea could be tested by comparing the ability to detect experimentally deployed threats by the two classes of individuals. Documenting survival is difficult. While we are confident that we could determine the fate of breeders with little error due to their sedentary nature, the Florida scrub-jay is long-lived, and the survival rate is high. Only a fraction, albeit non-negligible, of the breeders died during this study, perhaps reducing the power to detect an association between vigilance behaviour and survival.

## 5. Conclusions

The head-turning frequency in sentinels of the Florida scrub-jay is repeatable in breeders but not in juveniles or helpers and, therefore, likely becomes more repeatable as individuals age. This variation among individuals in vigilance behaviour was not related to survival during our study. Models of adaptive vigilance assume that investment in vigilance and the ways vigilance is achieved are under strong natural selection to detect predators efficiently [25]. Therefore, it is a concern that gazing strategies and survival were not correlated. Future studies are needed to validate this crucial assumption in animals.

## Figures and Tables

**Table 1:** Results from the bi-variate model in breeders examining the association between head-turning frequency in sentinels of the Florida scrub-jay and annual survival.

Bi-Variate Component	Variable	Mean Estimate	95% CI
Annual survival	Intercept	0.60	−2.79, 4.03
	Sex (male vs. female)	0.47	−0.47, 1.47
	Age	0.01	−2.71, 2.71
	Group size	0.46	−0.08, 1.10
Head-turning frequency	Intercept	42.31	40.55, 44.06
	Sex (male vs. female)	0.14	−1.76, 1.95
	Age	−0.35	−1.67, 1.02
	Group size	−1.87	−2.92, −0.81
	Height	−2.03	−3.03, −1.01
	Other sentinels (present vs. absent)	−3.05	−4.99, −1.07
Among-individual correlation	r	0.07	−0.92, 0.95

## Data Availability

The data are available from the corresponding author.
